# Nursing students’ patient safety competencies in the classroom and clinical settings: a cross-sectional study

**DOI:** 10.1186/s12912-024-01708-3

**Published:** 2024-01-17

**Authors:** Jamileh Farokhzadian, Gulcan Taskiran Eskici, Yasamin Molavi-Taleghani, Asghar Tavan, Hojjat Farahmandnia

**Affiliations:** 1 Nursing Research Center, Kerman University of Medical Sciences, Kerman, Iran; 2 Department of Nursing Management, Faculty of Health Sciences, Ondokuz Mayis University, Samsun, Turkey; 3 Health Management and Economics Research Center, Department of Health Services Management, School of Management and Medical Information Sciences, Isfahan University of Medical Sciences, Isfahan, Iran; 4 Health in Disasters and Emergencies Research Center, Institute for Futures Studies in Health, Kerman University of Medical Sciences, Kerman, Iran

**Keywords:** Competence, Classroom, Clinical, Nursing students, Patient safety

## Abstract

**Introduction:**

Patient safety is one of the critical indicators of providing qualified and high-quality health care services. Determining nursing students’ patient safety competencies will significantly contribute to the literature. Therefore, this study aimed to investigate Iranian nursing students’ patient safety competencies in classroom and clinical settings.

**Methods:**

In this cross-sectional study data were collected from 215 nursing of a university of medical sciences between February and May 2022, using a general questionnaire form and the Health Professional Education in Patient Safety Survey. Data analysis was done using descriptive and analytical statistics such as percentage, mean and paired-samples t-test.

**Results:**

The mean scores of nursing students’ the Health Professional Education in Patient Safety Survey were 3.50 ± 0.55 in the classroom and 3.45 ± 0.57 in the clinical setting. The highest mean scores of nursing students were in subdimension of “clinical safety” in both the clinical (3.91 ± 1.13) and classroom settings (3.91 ± 0.78). In addition, a statistically significant difference was found in patient safety learning confidence in the classroom versus clinical setting in the “culture of safety” subdimension.

**Conclusion:**

It appears that current educational programs provide opportunities to improve nursing students’ patient safety, but they are not enough. Nurse educators should apply new teaching methods and evaluate clinical strategies to meet educational needs.

## Background

In recent years, clinical risks have caused concerns and challenges throughout the healthcare process. Therefore, improving patient safety has been at the top of healthcare politics [1, 2]. This priority has drawn attention to healthcare professionals’ licensures and effectiveness of the nursing curriculum in preparing students with the appropriate patient safety competencies [3]. Nursing education has adopted a number of strategies and review processes to assess patient safety in nursing curricula in class and clinical settings [4, 5]. It is also possible to equip future nurses with competencies compatible with a modern patient safety [6, 7]. Patient safety competency is an individual’s ability to deliver a safe care in a given situation based on the care standards [8, 9]. Patient safety is the reduction of risk of unnecessary harm associated with healthcare to an acceptable minimum, an acceptable minimum refers to the collective notions of given current knowledge, resources available and the context in which care was delivered weighed against the risk of non-treatment or other treatment [10, 11].

Despite the emphasis on integrating patient safety into nursing education in international and national levels, studies have shown that nursing education does not address patient safety deeply and cannot bridge the gap between theory and practice [12–14]. Therefore, it is essential to enhance patient safety content in the current nursing programs by evaluating students’ competencies and perspectives on the content and teaching methods of patient safety [15, 16].

### Literature review

Literature review shows that a few studies have mainly examined the patient safety competencies of nursing students and their status in nursing programs. For example, Lukewich et al. reported that nursing students were relatively confident in what they were learning about the clinical dimensions of patient safety, but they were less confident about the sociocultural aspects of patient safety [17]. A systematic review study reported that the quality of the pedagogical atmosphere in the clinical settings had an important impact on the students’ overall level of competence. In this review, few studies describe the nursing students’ patient safety competencies and what they need to learn exactly [18]. Another systematic review highlighted the lack of research on patient safety in nursing education outlined areas in nursing education. In addition, the results of some studies addressed the lack of explicit integration of important elements of patient safety such as human factors into nursing curricula [19]. Furthermore, several studies evaluated patient safety competencies in the classroom and clinical settings. More efforts are required to embed deeply and consistently patient safety learning into health care education. Emerging changes in health professional education, ongoing studies to understand the extent of patient safety competencies among health professionals particularly at the entry into practice will be important [20, 21].

### Statement of the problem

Patient safety is a global concern that is part of health system programs in both developed and developing countries. Meanwhile, developing countries need to improve reliable program to reduce the consequences of not complying with patient safety principles [22]. Even with all of the attempts over the past two decades to minimize and avoid errors, new research indicates that one of the main worldwide factors of mortality and morbidity is unsafe care [23].

patient safety is also an emerging concern in Iranian healthcare education [24]. Limited Iranian literature examined undergraduate nursing students’ perspectives, knowledge, skills and attitude towards patient safety [13, 25, 26]. Limited evidence is available about how patient safety is addressed in nursing curricula and how students acquire patient safety competencies in both the classroom and clinical settings. Currently, we do not have much knowledge about the extent and nature of the role of nursing education in improving patient safety competencies. There is also limited evidence evaluating what competencies nursing undergraduates require or how well students are prepared to promote patient safety [13]. A comprehensive assessment of these topics can be helpful in improving educational programs for nursing students, Moreover, it can have suggestions for further evaluation of the nursing curriculum, especially in the courses related to clinical setting.

### The aim and questions of research

The objectives of this study are as follows: (a) to describe differences in nursing students’ self-reported patient safety competencies and (b) to compare their self-reported patient safety competencies in the classroom and clinical settings across academic years, (c) to investigate students’ perspectives on broader patient safety issues were addressed in health professional education, and understand how they were prepared for comfortable speaking up about patient safety.

The questions of this study are as follows:

- What are the nursing students learning about patient safety in the classroom and clinical settings based on the nursing curriculum?
- Which clinical learning environments could facilitate the development of patient safety competencies in nursing students.
- What are the students’ perceptions of broader aspects of patient safety and do they feel comfortable for talking about patient safety in both learning settings?

## Methods

### Study design and setting

This cross-sectional study was conducted from February to May 2022, at a large Nursing and Midwifery school affiliated with Kerman University of Medical Sciences. Kerman is the largest city in the southeast of Iran.

### Sample

All undergraduate nursing students who were studying in the second, fourth, sixth and eighth semesters (*N* = 381) were invited to participate in the study. Inclusion criteria were the nursing students, who had passed the “Fundamentals of Nursing Course”, started learning in the clinical settings and were not employed in a hospital. Incomplete questionnaire was considered as the exclusion criterion. Eligible participants included 350 students that were enrolled in the program. Research assistants handed out and distributed questionnaires to the participants in the Nursing and Midwifery school. We distributed 350 questionnaires, of which 72 were rejected due to incomplete completion of the tool and 63 questionnaires were not returned. Finally, data analysis was performed on (*n* = 215) participants. The overall response rate for inclusion in the analyses had to be 61.43%.

### Instruments and data collection

Data were collected using two questionnaires: a general characteristics questionnaire comprising the participants’ age, gender, attendance at patient safety training, observation of medical and nursing errors in clinical practices, experiences of reporting errors to clinical educators, hospital staff, and peer students, and questionnaire of Health Professional Education in Patient Safety Survey (H-PEPSS). The H-PEPSS is composed of 38 items divided into three sections. The first section deals with “learning about specific patient safety content areas” (27 items). This section is categorized into seven dimensions. (1) Clinical safety issues such as safe medication practices, hand hygiene, etc. (four items). These items are included in the H-PEPSS solely to help respondents distinguish between clinical and six socio-cultural dimensions of patient safety such as (2) working in teams (six items), (3) communicating effectively (three items), (4) managing safety risks (three items), (5) understanding human and environmental factors (three items), (6) Recognition, responsiveness to and disclose adverse events and close calls (four items), and (7) Culture of safety (four items). According to the nature of patient safety, which is both theoretical and practical, factors and items are reproduced for two different dimensions (classroom and clinical training): participants were asked to respond separately to each item regarding contents learned in the classroom and during their clinical experience. Therefore, mean scores of the dimensions were calculated for the classroom and clinical settings. The second section of the H-PEPSS (seven items) measures “How broader patient safety issues are addressed in health professional education”. The third section of the H-PEPSS with (four items) includes “comfortable speaking up about patient safety”. These sections were scored on a 5-point scale ranging from strongly disagree to strongly agree and included a “do not know” option. Higher scores of H-PEPSS indicate more perceptions of patient safety competency, broader aspects of patient safety and comfortable speaking up about patient safety [20, 21].

The content validity of the questionnaire was approved by 10 nursing faculty members in Kerman University of Medical Sciences. To assess the reliability of the questionnaire, 30 students of the seventh and eighth semesters, who were not involved in the study process, were asked to complete the questionnaire. Cronbach’s alpha coefficient was applied to assess reliability of each section. According to the results, Cronbach’s alpha coefficient ranged from 0.72 (clinical safety) to 0.83 (effective communication and safety risks management) for the class setting and from 0.78 (clinical safety) to 0.85 (working in teams) for the clinical setting. The internal consistency of the original H-PEPSS was re-evaluated using Cronbach’s alpha; the level of Cronbach’s alpha was between 0.81 and 0.85.

### Statistical analysis

The data were analyzed using SPSS version 21. General characteristics were summarized using descriptive statistics. Mean and standard deviation (SD) was calculated for each of the domain of the H-PEPSS and the means were compared. Moreover, all domains, the broader aspects of patient safety and comfortable speaking up were categorized into “strongly agree/agree” versus “neutral/disagree/strongly disagree” and frequency and percentage was calculated for each domain. The Kolmogorov Smirnov test showed that the data followed a normal distribution. The significance level was more than 0.05. So null hypothesize was accepted and parametric analytical tests were used. Paired t-tests were used to examine differences in confidence in patient safety learning in the class and clinical settings for each H-PEPSS dimension. We needed H-PEPSS developers and previous studies to analyze the instrument Statistical significance was considered *p* < 0.05.

## Results

### General characteristics information

Results showed that the majority of participants were below 25 years old, 89.8% were female, about 71.6% of the participants had no history of extracurricular and optional patient safety training and 88.8% observed medical and nursing errors in clinical practices, 78.1% reported errors to clinical educators, 40.9% reported errors to hospital staff, and 78.1% reported errors to peer students. Other general characteristics are shown in Table 1.

**Table 1:** General characteristics information of nursing students (*n* = 215)

Variables	Categories	n	%
Gender	Female	128	40.5
	Male	87	59.5
Age groups	< 25	193	89.8
	≤ 25	22	10.2
Attendance at patient safety training	Yes	61	28.4
	No	153	71.6
Observation of medical and nursing errors	Yes	191	88.8
	No	24	11.2
Reporting errors to educators	Yes	168	78.1
	No	47	21.9
Reporting errors to hospital staff	Yes	88	40.9
	No	127	59.1
Reporting errors to peer students	Yes	168	78.1
	No	47	21.9

### Comparing H-PEPSS domains and self-reported patient safety competency in different learning settings

In response to the first question of the study, the results demonstrated that nursing students’ total mean scores of patient safety competency were 3.45 ± 0.57, and 3.50 ± 0.55in clinical and classroom settings, respectively. Therefore, results showed that nursing students’ patient safety competency was partially higher than the average in both settings. In total, students reported lower levels of confidence in learning in the clinical settings in comparison with classroom settings.

Results showed that the highest mean scores of patient safety competency was related to “clinical safety” in both the clinical (3.91 ± 1.13) and classroom settings (3.91 ± 0.78). In the clinical settings, the lowest mean scores of patient safety competency were related to “culture of safety” (3.24 ± 0.92) and “understanding human and environmental factors” (3.24 ± 0.94). In the classroom settings, the lowest mean score of patient safety competency was related to “recognize, respond to and disclose adverse events and close calls” (3.27 ± 0.84).

Results showed that 84.65% of the nursing students chose “agree and strongly agree” for patient safety competency in the classroom, and 83.25% of them chose this option in the clinical settings. At domains level, 58.60% of the participants were confident that they learned “culture of safety” in the clinical setting, and 83.25% of them were confident that they learned “clinical safety” in the classroom.

In response to the second question of the study, Paired t test showed a statistically significant difference in the confidence in patient safety learning in the classroom versus clinical settings in “culture of safety” domain. There were no statistically significant differences in the confidence in patient safety learning in the classroom versus clinical settings in other H-PEPSS domains. The full information related to self-reported patient safety competency across the H-PEPSS domains was compared in the classroom and clinical settings and reported in Table 2.

**Table 2:** Classroom and clinical H-PEPSS domain scores for nursing students

Patient Safety Domains	Settings	N	Score (1–5)		Paired T-Test &	Agree/Strongly Agree (4–5 On Scale)		Kolmogorov–Smirnov test
			M	SD	*P*	N	%	KS Test (*P*-value)
Clinical Safety	Class	215	3.91	0.78	T = 0.01 *P* = 0.99	179	83.25	1.16 (0.13)
	Clinical	215	3.91	1.13		176	81.86	1.03 (0.23)
Working In Teams with Other Health Professionals	Class	215	3.28	0.82	T=-1.01 *P* = 0.31	142	66.04	1.24 (0.09)
	Clinical	215	3.33	0.79		155	72.09	1.27 (0.07)
Communicating Effectively	Class	215	3.65	0.92	T = 0.02 *P* = 0.97	167	77.67	1.18 (0.12)
	Clinical	215	3.65	0.84		165	76.74	1.21 (0.11)
Managing Safety Risks	Class	215	3.43	0.92	T=-0.38 *P* = 0.69	154	71.62	1.14 (0.14)
	Clinical	215	3.46	0.84		152	70.69	0.74 (0.62)
Understanding Human and Environmental Factors	Class	215	3.34	0.97	T = 1.61 *P* = 0.10	145	67.44	0.19 (0.51)
	Clinical	215	3.24	0.94		137	63.72	0.51 (0.79)
Recognize, Respond to And Disclose Adverse Events and Close Calls	Class	215	3.27	0.83	T = 0.67 *P* = 0.50	144	66.97	0.76 (0.59)
	Clinical	215	3.26	0.84		132	61.39	0.66 (0.71)
Culture Of Safety	Class	215	3.46	0.84	T = 3.62 *P* = 0.001	152	70.69	1.01 (0.20)
	Clinical		3.24	0.92		126	58.60	1.12 (0.16)
Overall Patient Safety Competency	Class	215	3.50	0.55	T = 1.71 *P* = 0.08	182	84.65	0.88 (0.21)
	Clinical	215	3.45	0.57		179	83.25	0.91 (0.18)

### Broader aspects of patient safety and comfortable speaking up about patient safety in both learning settings

In response to the third question of the study, the results showed that the total mean scores of the broader aspects of patient safety (3.66 ± 0.67) and comfortable speaking up about patient safety (3.31 ± 0.59) were above three (out of 5) in both learning settings. According to the median score of the scale (score 3), nursing students’ perceptions of all issues related to broader aspects of patient safety and comfortable speaking up about patient safety were at a moderate level in both clinical and class settings.

Results of this study from the viewpoint of students in both learning settings showed that 83.7% of the nursing students chose “agree and strongly agree” for the broader aspects of patient safety covered in their program and 64.7% of them chose this option for comfortable speaking up about patient safety. The full information related to broader aspects of patient safety and comfortable speaking up about patient safety in both learning settings showed in Table 3.

**Table 3:** Students’ perceptions on broader aspects of patient safety and comfort speaking up about patient safety

Broader aspects of patient safety:	N	M	SD	N	% Agree or strongly agree
As a student/trainee, my scope of practice was very clear to me	215	3.59	1	134	62.3
There is consistency in how patient safety issues were dealt with by different preceptors in the clinical/simulation setting	215	3.55	1.07	127	59.1
I had sufficient opportunity to learn and interact with members of interdisciplinary teams	215	3.58	0.96	122	56.7
I gained a solid understanding that reporting adverse events and close calls can lead to change and can reduce reoccurrence of events	215	3.83	0.87	144	67
Patient safety was well integrated into the overall program	215	3.64	1	127	59.1
Clinical aspects of patient safety (e.g., hand hygiene, transferring patients, medication safety) were well covered in our program	215	3.78	1	142	66
“System” aspects of patient safety were well covered in our program (e.g., aspects of the organization, management, or the work environment including policies, resources, communication and other processes)	215	3.59	0.94	116	54
**Total**	215	3.66	0.67	180	83.7
**Comfort speaking up about patient safety**:	**N**	**M**	**SD**	**N**	**% Agree or strongly agree**
If I see someone engaging in unsafe care practice in the clinical setting, I feel I can approach them	215	3.49	1.01	127	59.1
If I make a serious error, I worry that I will face disciplinary action	215	3.57	1.09	128	59.5
It is difficult to question the decisions or actions of those with more authority	215	3.05	1.05	77	35.8
In clinical/simulation settings, discussion around adverse events focuses mainly on system-related issues, rather than focusing on the individual(s) most responsible for the event	215	3.12	1.05	80	37.2
**Total**	215	3.31	0.59	139	64.7

## Discussion

In this study nursing students reported lower levels of confidence in learning in the clinical setting in comparison with the classroom setting. In a similar study in Jordan, it was shown that the patient safety competence of nursing students was better in the field of knowledge than in the clinical field, which was in line with the results of our study. It was also shown that students in advanced years were less confident about their patient safety knowledge and competencies than students in previous years [27]. In the study of Vaismoradi et al., was shown that from the point of view of nursing students, it is much more possible to improve knowledge and performance related to patient safety in the clinical environment [13].In a study with the same purpose as our study in Saudi Arabia, it was shown that in many aspects of patient safety, nursing students reported a higher score in the classroom environment, which is not consistent with the results of our study. It can be said that there may be factors such as the difference in educational strategies or depending on whether the focus of educational programs is on the classroom or on the clinical environment, these results may be different [1].

It should also be noted that two aspects of classroom training and clinical environment together will lead to patient safety competence in nursing students. With less attention to each of these two educational environments, the desired result will not be achieved [28].

The results demonstrated that the highest mean score of patient safety competency was related to the domain of “clinical safety” in the clinical and classroom settings. The results of other studies are consistent with our results and have reported the “clinical safety” dimension as the highest score of the patient safety dimension [27, 29–31].. It seems that this dimension can be considered a successful basis in classroom and clinical training programs.

The findings of the study demonstrated the lowest scores of patient safety competency in the clinical setting were related to the domains of “understanding human and environmental factors” and “culture of safety” respectively. This result was consistent with studies of [30, 32] and inconsistent with Doyle. et al. study [29]. These results suggest that the safety culture should be improved in the clinical setting. In addition, different culture of the clinical setting in Iran may reduce nursing students’ understanding of the human and environmental factors. Concerning the low score of “understanding human and environmental factors”, it can be declared that there is a poor interaction between nursing educators and students, and nursing staff that should be improved.

Our finding showed that the lowest score of patient safety competency in the classroom setting was related to the domain of “recognize, respond to and disclose adverse events and close calls”. This result was consistent with results of other studies [13, 33]. It can be declared that the students may not get enough theoretical information from the teacher in the classroom setting. It is suggested to carry out more investigations and studies related to this dimension in nursing education programs.

The results showed that 80% of the nursing students chose “agree and strongly agree” for all dominoes of patient safety competency in both classroom and clinical settings. similar results were recorded in studies Mansour and Lee et al. [6, 34]. This positive approach of the student toward domains of patient safety competency is a matter that show base of Iranian nursing curriculum educational contents of all dominoes of patient safety provided by educators in both settings is identical.

The results showed a statistically significant difference in the confidence in patient safety learning in the class versus clinical settings in “culture of safety” domains. The results demonstrate a gap between theory and practice in nursing curriculum. This result was consistent with several studies [35, 36] that could be explained by factors such as variations in clinical instructor’s attitudes and insights into clinical setting compared with classroom setting and also students’ experiences across a variety of different educational hospitals that can influence on the nursing students’ perspectives related to culture of safety.

In this study showed that nursing students’ perceptions of all issues related to broader aspects of patient safety and comfortable speaking up about patient safety were at moderate levels in both clinical and class settings. This result was consistent with previous studies [17, 30, 37]. Therefore, classroom educators and clinical educators should adopt role-modeling behaviors and encourage nursing students to raise concerns about patient safety. A patient safety course such as “world health organization (WHO) Patient safety curriculum guide: multi-professional edition” should be developed and integrated into the nursing syllabus to improve nursing students’ perspective in related to broader aspects of patient safety and comfortable speaking up about patient safety and to address important patient safety concepts.

### Limitations

This study had several limitations: The first limitation was individual judgments of the nursing students when completing the study questionnaire, level of their commitment and responsibility in both clinical and classroom settings and the amount of training and support received from educators, as well as variables that influenced on the nursing students’ perspective of patient safety and our study did not investigate these variables. The second limitation was the use of self-report scales because the respondents might have refused to answer the questions honestly. This limitation was partially overcome by communicating to the participants properly and explaining that their participation is optional, their responses will be kept confidential, and they can fill it without writing their names on it. The third limitation was the non-use of students’ practical competencies in the clinical environment, which was not possible due to the non-availability of specific tools.

## Conclusion

The results provided a clear understanding of the status of patient safety competency among undergraduate nursing students in classroom and clinical settings. Overall, Iranian nursing students reported moderate patient safety competencies. In our study, nursing students declared that learning of various aspects of patient safety competencies was different in classroom and clinical settings, so that they did not learn much in the clinical setting. Therefore, the gap between theory and practice in patient safety education is felt more. If the classroom and the clinical setting educator are two separate individuals, they should coordinate with each other regarding the design, implementation, and evaluation of the educational program. It appears that current educational programs provide opportunity to improve nursing students’ patient safety but they are not enough. Revising nursing curriculum, applying new teaching methods, and clinical evaluation strategies are suggested to meet professional needs. A qualitative study should be designed in the future to explore the perspectives of both nursing students and faculty members regarding patient safety content especially in clinical settings.

## Data Availability

The data sets generated during the current study are available from the corresponding author.

## References

[1] Colet PC (2015). Patient safety competence of nursing students in Saudi Arabia: a self-reported survey. Int J Health Sci.

[2] Kiesewetter J (2014). German undergraduate medical students’ attitudes and needs regarding medical errors and patient safety–a national survey in Germany. Med Teach.

[3] Abu-El-Noor NI (2019). Patient safety culture among nurses working in Palestinian governmental hospital: a pathway to a new policy. BMC Health Serv Res.

[4] VanDenKerkhof E (2017). Patient safety in practical nurses’ education: a cross-sectional survey of newly registered practical nurses in Canada. Nurse Educ Today.

[5] Topaz M (2016). Nurses’ perspectives on patient satisfaction and expectations: an International Cross-sectional Multicenter Study with implications for evidence-based practice. Worldviews Evid Based Nurs.

[6] Mansour M (2012). Current assessment of patient safety education. Br J Nurs.

[7] Bishop AC, Macdonald M (2017). Patient involvement in Patient Safety: a qualitative study of nursing staff and patient perceptions. J Patient Saf.

[8] Almaramhy H (2011). Knowledge and attitude towards patient safety among a group of undergraduate medical students in Saudi Arabia. Int J Health Sci (Qassim).

[9] Bridges JF, Loukanova S, Carrera P. *Patient empowerment in health care* 2008.

[10] Abbott JF (2020). To the point: integrating Patient Safety Education into the obstetrics and Gynecology Undergraduate Curriculum. J Patient Saf.

[11] Giap TT, Park M. Implementing patient and family involvement interventions for promoting patient safety: a systematic review and Meta-analysis. J Patient Saf; 2020. 10.1097/PTS.0000000000000714 33208637

[12] Alquwez N (2018). Nurses’ perceptions of patient safety culture in three hospitals in Saudi Arabia. J Nurs Scholarsh.

[13] Vaismoradi M, Salsali M, Marck P (2011). Patient safety: nursing students’ perspectives and the role of nursing education to provide safe care. Int Nurs Rev.

[14] Stevanin S (2015). Knowledge and competence with patient safety as perceived by nursing students: the findings of a cross-sectional study. Nurse Educ Today.

[15] Nancy-Jane Smith B (2017). Self-Assessment of Patient Safety competence: a Questionnaire Survey of Final Year British and Finnish pre-registration nursing students. Int J Caring Sci.

[16] Ammouri A (2015). Patient safety culture among nurses. Int Nurs Rev.

[17] Lukewich J (2015). Undergraduate baccalaureate nursing students’ self-reported confidence in learning about patient safety in the classroom and clinical settings: an annual cross-sectional study (2010–2013). Int J Nurs Stud.

[18] Bianchi M (2016). Patient safety competencies in undergraduate nursing students: a rapid evidence assessment. J Adv Nurs.

[19] Okuyama A, Martowirono K, Bijnen B (2011). Assessing the patient safety competencies of healthcare professionals: a systematic review. BMJ Qual Saf.

[20] Ginsburg L (2012). The H-PEPSS: an instrument to measure health professionals’ perceptions of patient safety competence at entry into practice. BMJ Qual Saf.

[21] Ginsburg LR, Tregunno D, Norton PG. *Self-reported patient safety competence among new graduates in medicine, nursing and pharmacy*. BMJ Qual Saf, 2012: p. bmjqs–2012. 10.1136/bmjqs-2012-001308 PMC3585493 23178859

[22] Organization WH. Global patient safety action plan 2021–2030: towards eliminating avoidable harm in health care. World Health Organization; 2021.

[23] Organization WH. *Facts on patient safety* 2014. Retrieved from website: http://www.who.int/features/factfiles/patient_safety/patient_safety_facts/en/index.html.

[24] Azami-Aghdash S (2015). Patient safety culture in hospitals of Iran: a systematic review and meta-analysis. Med J Islamic Repub Iran.

[25] Safarpour H (2017). Patient safety attitudes, skills, knowledge and barriers related to reporting medical errors by nursing students. Int J Clin Med.

[26] Nabilou B, Feizi A, Seyedin H (2015). Patient safety in medical education: students’ perceptions, knowledge and attitudes. PLoS ONE.

[27] Suliman M (2019). Measuring patient safety competence among nursing students in the classroom and clinical settings. Nurs Educ Perspect.

[28] Panimdim CY, Ismael JD. *The correlation of academic and clinical performance among nursing students of batch 2015–2017*. in *Proceedings of the* 2018 *2nd International Conference on Education and E-Learning*. 2018.

[29] Huang FF (2020). Self-reported confidence in patient safety competencies among Chinese nursing students: a multi-site cross-sectional survey. BMC Med Educ.

[30] Doyle P (2015). Self-reported patient safety competence among Canadian medical students and postgraduate trainees: a cross-sectional survey. BMJ Qual Saf.

[31] Ginsburg LR, Tregunno D, Norton PG (2013). Self-reported patient safety competence among new graduates in medicine, nursing and pharmacy. BMJ Qual Saf.

[32] Del Prato D (2013). Students’ voices: the lived experience of faculty incivility as a barrier to professional formation in associate degree nursing education. Nurse Educ Today.

[33] Vaismoradi M (2014). Nursing students’ perspectives of the cause of medication errors. Nurse Educ Today.

[34] Lee NJ, Jang H, Park SY (2016). Patient safety education and baccalaureate nursing students’ patient safety competency: a cross-sectional study. Nurs Health Sci.

[35] Langari MNM (2017). Self-assessment of patient safety competence: a questionnaire survey of final year British and Finnish pre-registration nursing students. Int J Caring Sci.

[36] Tella S (2015). Learning to ensure patient safety in clinical settings: comparing Finnish and British nursing students’ perceptions. J Clin Nurs.

[37] Raymond JM, Medves JM, Godfrey CM (2017). Baccalaureate nursing students’ confidence on patient safety. J Nurs Educ Pract.

